# Cardiovascular Diseases in Sub-Saharan Africa Compared to High-Income Countries: An Epidemiological Perspective

**DOI:** 10.5334/gh.403

**Published:** 2020-02-12

**Authors:** Matthew Fomonyuy Yuyun, Karen Sliwa, Andre Pascal Kengne, Ana Olga Mocumbi, Gene Bukhman

**Affiliations:** 1 Department of Medicine, Harvard Medical School, Boston, US; 2 Cardiology and Vascular Medicine Service, VA Boston Healthcare System, Boston, US; 3 Hatter Institute for Cardiovascular Research in Africa, University of Cape Town, ZA; 4 South African Medical Research Council and Department of Medicine, University of Cape Town, ZA; 5 Instituto Nacional de Saúde and Universidade Eduardo Mondlane, Maputo, MZ; 6 Division of Cardiovascular Medicine, Brigham and Women’s Hospital, Boston, US

**Keywords:** cardiovascular diseases, risk factors, sub-Saharan Africa, high-income countries, non-communicable diseases

## Abstract

Non-communicable diseases (NCDs) are the second common cause of death in sub-Saharan Africa (SSA) accounting for about 35% of all deaths, after a composite of communicable, maternal, neonatal, and nutritional diseases. Despite prior perception of low NCDs mortality rates, current evidence suggests that SSA is now at the dawn of the epidemiological transition with contemporary double burden of disease from NCDs and communicable diseases. In SSA, cardiovascular diseases (CVDs) are the most frequent causes of NCDs deaths, responsible for approximately 13% of all deaths and 37% of all NCDs deaths. Although ischemic heart disease (IHD) has been identified as the leading cause of CVDs mortality in SSA followed by stroke and hypertensive heart disease from statistical models, real field data suggest IHD rates are still relatively low. The neglected endemic CVDs of SSA such as endomyocardial fibrosis and rheumatic heart disease as well as congenital heart diseases remain unconquered. While the underlying aetiology of heart failure among adults in high-income countries (HIC) is IHD, in SSA the leading causes are hypertensive heart disease, cardiomyopathy, rheumatic heart disease, and congenital heart diseases. Of concern is the tendency of CVDs to occur at younger ages in SSA populations, approximately two decades earlier compared to HIC. Obstacles hampering primary and secondary prevention of CVDs in SSA include insufficient health care systems and infrastructure, scarcity of cardiac professionals, skewed budget allocation and disproportionate prioritization away from NCDs, high cost of cardiac treatments and interventions coupled with rarity of health insurance systems. This review gives an overview of the descriptive epidemiology of CVDs in SSA, while contrasting with the HIC and highlighting impediments to their management and making recommendations.

**Highlights:**

– The burden of non-communicable diseases including cardiovascular diseases is rising in SSA.
– Levels of hypertension diagnosis, treatment, and control are low at <40%, <35%, and 10–20%, respectively, and more than 40% of patients with diabetes are not aware of their diagnosis in SSA.
– SSA has 23% of the world’s prevalent rheumatic heart disease cases.
– The leading causes of heart failure in SSA are hypertensive heart disease, cardiomyopathy, and rheumatic heart disease, with ischemic heart disease accounting for <10% of cases compared to >50% in high-income countries.

## Introduction

Worldwide, non-communicable diseases (NCDs) are the leading cause of death, accounting for 73.4% of all deaths, led by cardiovascular diseases (CVDs), with ischemic heart disease (IHD) being the most frequent cause of cardiovascular death in the 2017 Global Burden of Disease (GBD) study [1]. NCDs are the second most common cause of death in sub-Saharan Africa (SSA) accounting for 2.6 million deaths, equivalent to about 35% of all deaths, after a composite of communicable, maternal, neonatal, and nutritional diseases (CMNNDs). However, the prevalence, incidence and mortality rates of CMNNDs peaked in the late 1990s and early 2000s, and their contributions to overall mortality have been progressively declining, though still extremely frequent [2,3]. There is a contemporary double burden of disease from NCDs and communicable diseases. However, in an optimistic projection scenario of a continuous fall in CMNNDs deaths, NCDs are now projected to account for more than half all deaths by 2030 in SSA [4]. In high-income countries (HIC) of Western Europe and North America, where the epidemiologic transition occurred around the beginning to middle of the 20th century, NCDs are the leading cause of morbidity and mortality and account for 90% of all deaths, with the leading NCDs being CVDs [2].

Although age-adjusted CVD mortality rates in SSA are low compared to HIC, absolute number of CVD deaths has increased by more than 50% in the past three decades in this region [2] and tend to occur at younger ages, leading to a high number of disability adjusted life years (DALYs) [5,6,7,8,9,10,11,12]. IHD, stroke, and hypertensive heart disease are the three most common causes of CVD death in SSA [2,13]. Concurrently, the neglected endemic CVDs of SSA, such as endomyocardial fibrosis and rheumatic heart disease as well as congenital heart diseases, remain unconquered [14,15,16,17]. Nevertheless, the growth of cardiac care infrastructure and professionals in SSA have failed to match up with the growing burden and demands of these diseases [18,19,20]. Given mounting concerns about the growing burden of NCDs globally, the 2011 UN High-Level Meeting made a political declaration on non-communicable diseases aimed at achieving the goal of a 25% reduction in premature NCD mortality by 2025 (the 25 by 25 goal), which was further emphasized in the UN Sustainable Development Goals (SDGs) for 2030 adopted in 2015 to reduce by one third premature mortality from NCDs by 2030 [21]. Recent studies suggest that Africa is not on track for achieving these NCD targets and indicators by the set deadlines [22]. This critical review gives a descriptive epidemiology and risks factors of specific CVDs in SSA, highlights areas where urgent action needs to be undertaken, and makes some substantive recommendations. HIC throughout this review will be referring to Western Europe and North America.

## Methods

We searched PUBMED/Medline and conducted manual searches from the bibliographies of relevant articles for papers on cardiovascular diseases, non-communicable diseases, and various cardiovascular disease entities in sub-Saharan Africa. The search was restricted to published English-language articles from January 1990 to March 2019, and used a combination of MeSH terms and keywords related to coronary artery disease or ischemic heart disease, stroke or cerebrovascular disease, hypertensive heart disease, heart failure, rheumatic heart disease, valvular heart disease, congenital heart disease, endomyocardial fibrosis, pulmonary hypertension, pericarditis, HIV and cardiovascular diseases, aortic aneurysms, and peripheral vascular disease in Africa, from which sub-Saharan African studies were identified. One example of a search term was ‘Coronary artery disease OR ischemic heart disease AND Africa’. The identified articles were screened (title, abstract, and full text as appropriate), and the most relevant were included in the current review. We then organized our results into *a priori* identified themes: hospital-based, community-based, incidence, prevalence, risk factors, diagnosis, management, and mortality and other outcomes. Each study identified in our search strategy was then tabulated according to theme and findings were synthesized to construct a narrative review. This is not a systematic review.

## Classical CVD Risk Factors

Traditional modifiable atherothrombotic CVD risk factors of smoking, hypertension, diabetes, hyperlipidemia, obesity, physical inactivity, and poor dietary habits identified from HIC populations are now palpably emergent in SSA [13,23,24]. However, the true burden of these risk factors, and their ensuing complications in SSA remain uncertain as most countries are either void of data or have deficient data collection systems that are not sufficiently reliable to enable mounting of a commensurate health-system response [25]. Table 1 depicts the prevalence rates of these CVD risk factors in SSA, compared to HIC of the western world and globally. Smoking rates are 10% in SSA versus 30% in HIC [13,26,27]; hypertension prevalence in individuals ≥18 years old is 30% in SSA (40% in urban and 20% in rural populations) versus 20% in HIC [28,29,30]; diabetes prevalence in ≥18 years old is 7.1% in men and women in SSA compared to 6–8% in men and 3–6% in women in HIC [25,31]; dyslipidemia prevalence in adults is 25% in SSA versus 40–60% in HIC [32,33,34,35,36,37]; physical inactivity prevalence is 22% in SSA versus 29–40% in HIC [38,39,40]; and obesity whose prevalence rates are variable in SSA and higher among women (2–40%) compared to men (1–15%) versus 18–35% in women and 12–30% among men in HIC [34,41,42].

**Table 1:** Cardiovascular risk factors prevalence in Sub-Saharan Africa compared to high-income countries and worldwide.

CVD risk factor	SSA	Western Europe	North America	Global

**Smoking in ≥15 years of age [13,26,27]**	– Total 10% – Males 18% – Females 2.3%	Total 30% – Males 38% – Females 21%	Total 14–21% – Males 16–24% – Females 11–18%	Total 15% – Males 25% – Females 5%
**Hypertension in adults ≥18 years old [28,29,30]**	– 30s%* – <40% are aware of their diagnosis – <35% on treatment – <10–20% controlled HTN	– 20s%* – 60–70% aware of their diagnosis – 50% on treatment – 30–40% controlled HTN	– 20s%* – >80% aware of their diagnosis – >70% on treatment – 55–66% controlled HTN	– Men 24%* – Women 20%*
**Diabetes Mellitus [2,25,31]**	*GBD (all ages):* – Total 3.5% – Men 3.7% – Women 3.3%	*GBD (all ages):* – Total 10.2% – Men 11.1% – Women 9.3%	*GBD (all ages):* – Total 9.9% – Men 10.9% – Women 8.9%	*GBD (all ages):* – Total 6.5% – Men 6.7% – Women 6.2%
	*NCD RFC* ≥ *18 years old:* – Men 7.1% – Women 7.1%	*NCD RFC* ≥ *18 years old:* – Men 6–8% – Women 3–6%	*NCD RFC* ≥ *18 years old:* – Men 6–8% – Women 5–6%	*NCD RFC* ≥*18 years old:* – Total 8.5% – Men 9.0% – Women 7.9%
**Hyperlipidaemia ≥18–≥20 years old [32,33,34,35,36,37]**	– Total 25% – High risk ~ 40%	– Total 40–60% – High risk ~ 70%	– Total 40–45 % – High risk ~ 70%	– Total 39% – Men 37% – Women 40%
**Physical inactivity [38,39,40]**	– Total 22% – Men 18% – Women 26%	– Total 29% – Men 26% – Women 32%	– Total 30–40 % – Men 25–31% – Women 31–41%	– Total 27% – Men 23% – Women 32%
**Obesity [34,41,42]**	– Men 1–15% – Women 2–40%	– Men 12–22% – Women 18–25%	– Men 20–30% – Women 21–35%	– 12% of all adults

* Age-standardised prevalence; SSA: Sub-Saharan Africa; GBD: Global Burden of Disease (2017); HTN: hypertension; NCD RFC: Non-communicable disease Risk Factor Collaboration systematic review (2014). North America here is USA & Canada.

The burden of tobacco-related mortality in Africa increased by about 70% from 1990 to 2016 [43]. Of great concern is the fact that levels of hypertension diagnosis, treatment, and control remain <40%, <35%, and 10–20%, respectively [44,45,46]. These figures are very troubling when compared to the North America where >80% of patients are aware of their diagnosis, >70% are receiving treatment, and >55% have adequate blood pressure control [34,47], and corresponding figures for Western Europe are of 60–70%, 50%, and 30–40% respectively [47]. In SSA, diabetes is still undiagnosed in a high proportion of the population, as more than 40% of patients with diabetes are not aware of their diagnosis, and less than half of patients on medication have well-controlled diabetes [23]. Although SSA is a latecomer to the obesity epidemic compared to the HIC, the prevalence in this region is rapidly rising. This problem might be compounded by anecdotal and evidence-based reports that in some SSA regions, overweight and obesity are the preferred body images and are still regarded admirably as indicators of health, affluence, well-being and happiness, especially among women [48]. Overall, it is important to recognize the enormous contributions from several studies on risk factors in many countries, leading to significant advances in last 20 years in SSA.

## Cardiovacular Diseases

Table 2 depicts causes of cardiovascular deaths in decreasing order of frequency in SSA, the western world, and worldwide in 2017.

**Table 2:** Causes of cardiovascular death including congenital heart disease in decreasing order in sub-Saharan Africa, high-income countries, and worldwide in 2017.

SSA		Western Europe		North America		Global	

CVDs	Number (%)	CVDs	Number (%)	CVDs	Number (%)	CVDs	Number (%)

All CVDs	940,991 (100%)	All CVDs	1,356970 (100%)	All CVDs	986,759 (100%)	All CVDs	18,052,195 (100%)
IHD	369,526 (39.26%)	IHD	662,226 (48.80%)	IHD	581,609 (58.94%)	IHD	8,930,369 (49.47%)
Stroke	317,766 (33.76%)	Stroke	330,431 (24.35%)	Stroke	190,423 (19.30%)	Stroke	6,167,291 (34.16%)
HHD	71,179 (7.56%)	HHD	74,726 (5.51%)	HHD	44,682 (4.53%)	HHD	925,675 (5.13%)
CHD	66,879 (7.12%)	AF/AFL	67,015 (4.94%)	AF/AFL	34,532 (3.50%)	CM	368,535 (2.04%)
CM	24,304 (2.58%)	NRVHD	46,288 (3.41%)	CM	31,827 (3.23%)	AF/AFL	287,241 (1.59%)
RHD	15,920 (1.69%)	CM	43,621 (3.21%)	NRVHD	24,323 (2.46%)	RHD	285,517 (1.58%)
AF/AFL	10,372 (1.10%)	AA	30,904 (2.28%)	PAD	17,420 (1.76%)	CHD	261,247 (1.45%)
Endocarditis	8775 (0.93%)	PAD	17,420 (1.28%)	AA	14,992 (1.52%)	AA	167,248 (0.93%)
AA	7,708 (0.82%)	RHD	17,336 (1.28%)	RHD	11,894 (1.21%)	NRVHD	144,859 (0.80%)
NRVHD	5,114 (0.54%)	Endocarditis	15,025 (1.11%)	Endocarditis	10,140 (1.02%)	Endocarditis	83,390 (0.46%)
PAD	1,951 (0.21%)	CHD	2,538 (0.19%)	CHD	3,522 (0.36%)	PAD	70,168 (0.39%)
Other CVDs	41,492 (4.46%)	Other CVDs	48,258 (3.64%)	Other CVDs	21,390 (2.17%)	Other CVDs	360,650 (2.00%)

AA (aortic aneurysms); AF/AFL (atrial fibrillation/flutter); IHD (ischemic heart disease); CHD (congenital heart disease); CM (cardiomyopathy); CVDs (cardiovascular diseases): HHD (hypertensive heart disease); NRVHD (non-rheumatic valvular heart disease); PAD (peripheral artery disease); RHD (rheumatic heart disease); SSA (Sub-Saharan Africa). Table shows number of deaths from specific CVDs aetiologies and percentage contribution of each to total CVDs deaths. North America here is USA & Canada. Adapted from Global Burden of Diseases 2017 [2].

### Ischemic Heart Disease

Ischemic heart disease (IHD) is the leading cause of death globally, responsible for about 9 million deaths (16% of all deaths) and accounting for 50% of all cardiovascular deaths worldwide in 2017 [1]. With epidemiologic transition in the western world and other high-income countries, this increasing trend in mortality from IHD was noted to have begun as early as the 1920s and peaked in the late 1960s and 1970s and then started to decline in the 1980s, while mortality increased in other world regions before attenuating after the dawn of the 21st century [49,50]. Historically, very low prevalence, incidence and mortality from IHD have been observed in SSA compared to the HIC [51,52,53,54]. Back in the 1990s it was noted to account only for 6% of all CVDs in studied SSA regions [55]. Recent data from the Global Burden of Disease Study in 2017 revealed that IHD is now the most frequent cause of CVD death in SSA (5% of all deaths, 40% of CVDs deaths), followed by stroke. However, the Global Burden Disease Study derives some of its findings from statistical modelling tools used as a result of the scarcity of data in some regions. There is therefore a possibility that the high rate of IHD mortality seen in this study is an overestimate for SSA, as data from few studies in the field suggest relatively low rates of IHD in SSA [7,53,56,57]. In high-income countries, IHD is the most frequent cause of CVD death (16–19% of all deaths, 50–60% of CVD deaths) [2].

Earlier studies on IHD in SSA revealed the rarity of this disease from the 1940s to 1990s with rates of 0.3–3% on various study populations, though a steady rise was observed [51,52,54]. Among the world’s poorest billion people (majority in SSA), IHD was found to account for only a relatively low 12% of the CVD DALYs, compared with 51% of DALYs in HIC [58]. Nevertheless, in the 2000s it was noted that IHD measures and its risks factors were beginning to rise in SSA [5,11,59,60], with mortality tending to occur at relatively younger ages when compared to high-income regions [11,50,61,62]. Between 1990 and 2017, the number of deaths from IHD in SSA witnessed a crude percentage increase of 74% [2]. Most importantly, there is accruing evidence suggesting that low IHD rates in SSA might not be real, but rather related to poor ascertainment, and that IHD may be underdiagnosed due to paucity of biomarker, electrocardiography, invasive angiography, and diagnostic imaging capabilities [63,64]. Interventions aimed at primary prevention of IHD through risk factor reduction in SSA have been fruitful [65].

### Stroke (Cerebrovascular Disease)

Globally, in 2017, there were 6.2 million deaths from stroke accounting for 11% of all deaths and ~35% of all CVD deaths, the second most common cause of cardiovascular death after IHD. Stroke was the second most frequent cause of CVD death in SSA (4% of all deaths, 34% of CVD deaths), and HIC (6–8% of all deaths, 19–24% of CVD deaths) in the most recent GBD study [1]. However, most other reports have identified stroke as the most frequent cause of CVD death in SSA [5,11]. In SSA, ischemic stroke is more incident (55% of all incident strokes) and more prevalent (63% of all prevalent strokes), but haemorrhagic [66] stroke is more fatal (65% of all stroke fatalities). Ischemic stroke accounts for 55% of all stroke deaths in western Europe and 46% of North America [2]. In the INTERSTROKE study, 70% of strokes in SSA were due to ischemic stroke and 30% due to hemorrhagic stroke, compared to 93.3 % and 6.7% respectively in the HIC of the western world [66]. There is a relatively large percentage of haemorrhagic strokes in SSA mainly due to hypertension when compared to high-income countries, with very high mortality and happening 10 to 15 years earlier than what happens in the developed countries [11,66,67].

The number of deaths from stroke in SSA have increased by nearly 50% in past three decades [2,11,68]. Stroke tends to affect the younger productive workforce in SSA compared to the developed world [6], and the mean age at death from stroke in SSA also the lowest among all low- to middle-income countries [2,11,66]. Community-based studies in Africa have revealed age-standardized (to the WHO world population) annual stroke incidence rates of up to 316 per 100,000 population, and age-standardized prevalence rates of up to 981 per 100,000 [67,68]. Stroke case-fatality rates in SSA are the highest in the world ranging from 21–47% in various studies [66,68,69], compared to 17–30% in high-income countries [70], and almost double for hemorrhagic stroke compared to ischemic stroke [67,68]. A few studies have demonstrated overall positive patient outcomes following acute stroke care interventions in Africa [71]. Given that hypertension is the single most important preventable risk factor for stroke in SSA [6,66], its optimal control should be the main focus to help reduce stroke occurrence.

### Heart Failure

Population-based incidence, prevalence, and mortality studies on heart failure are lacking in SSA, but it is likely to affect millions in this region. Case fatality rates are high with estimated six-month mortality from heart failure in SSA being 18% [7]. The prevalence of heart failure among adults in HIC (Western Europe, Canada, USA) is approximately 1–2%, and increasing with age to more than 10% in people aged 70 and above [34,72,73]. Despite improvements in survival, absolute mortality rates from heart failure remain approximately 50% within five years of diagnosis and 30-day heart failure re-hospitalization rates are still about 25% in developed countries [73]. While the underlying etiology of heart failure in adults in HIC is IHD [34,74,75], in SSA the leading causes are hypertensive heart disease, cardiomyopathy, and rheumatic heart disease [7,9,53,56,76,77], although the relative percentage of heart failure caused by IHD in SSA in increasing [57]. In fact, hypertensive heart disease is the most common cause of *de novo* heart failure presentations in adults in SSA [7,9,53,56,57,76,77]. In a worldwide systematic review of heart failure aetiologies, an overlap of underlying aetiologies was observed, and crude IHD prevalence was >50% in Europe and North America and <10% in SSA [76]. The most common etiologies of heart failure among children in SSA are congenital heart disease (52%) and rheumatic heart disease (36%) in one study [9].

A recent meta-analysis of studies in SSA depicted that hypertensive heart disease (39%) was the commonest cause of HF in SSA, followed by cardiomyopathies (21%) and rheumatic heart disease (14%), with ischemic heart disease less frequent (7%) [56]. In the THESUS–HF study in 9 SSA countries, heart failure was most commonly due to hypertension (45%), followed by rheumatic heart disease (14%), while ischemic heart disease accounted for a lower percentage (~8%) [7]. The THESUS-HF registries findings differ from North American (ADHERE) [75] and European (EHFS II) [74] registries where ischemic heart disease was the etiology of acute decompensated heart failure in 57% and 54% respectively [78].

Particular aspects of heart failure epidemiology in SSA include high incidence of peripartum cardiomyopathy and right heart failure, as well as its occurrence at an early age, compared to HIC. Peripartum cardiomyopathy is an important cause of heart failure in SSA with incidence as high as 1:100 life-births in women in Nigeria and 1:1000 in South Africa compared to 1:1100 to 1:4000 in USA (where it is higher among African American women) [79,80], and accounted for 7.7% of all acute heart failure cases in a large SSA registry [7]. One notable difference between newly diagnosed heart failure presentations between the HIC is the finding of nearly 30% right heart failure (RHF) observed in one country in SSA (South Africa) [81] compared to <5% of RHF cases in Europe [74]. Acute heart failure occurs at a relatively young mean age in the fifties in SSA, compared to developed countries where it is a disease of the elderly, with a mean age in the seventies [74,75], thus presenting about two decades earlier in SSA [7].

### Rheumatic Heart Disease

Rheumatic valvular disease (RHD) is the most significant valvular disease, with an age-standardized death rate of 3.7/100,000 worldwide in 2017 compared to 2.0/100,000 from non-rheumatic valvular heart disease [2]. Globally, RHD is the most common acquired cardiovascular disease in young people <25 years of age [8]. SSA has 23% of the world’s prevalent RHD cases (8.9 million of world’s 39.3 million estimated cases in 2017 were in SSA), with high prevalence rate of 864/100,000 compared to only 9.8 and 7.7/100,000 in North America and Western Europe respectively [2]. In SSA, RHD is the second most common cause of heart failure in children after congenital heart disease and third most common etiology of heart failure in adults after hypertension and dilated cardiomyopathies [9,53,56,57]. Nonetheless, disease management remains sub-optimal and case fatality high in SSA and other low- and middle-income countries [17]. In the REMEDY study on RHD which involved 12 countries from Africa (with exception of Egypt, all were from SSA), Yemen and India, patients were young (median age, 28 years), only 55% of patients were on secondary antibiotic prophylaxis, and despite the fact that 69.5% of patients with clinical indication of oral anticoagulation were prescribed one, only 28.3% had a therapeutic international normalized ratio; only 3.6% of women of child-bearing age were on contraception. Two-year case fatality rate was high, at 16.9%, with median age at death at 29 years [17]. Arrival of the immigrant population from poorer countries, where RHD is endemic to HIC might be responsible for a new burden of RHD in the western world [8].

### Non-Rheumatic Valvular Heart Disease

Non-rheumatic valvular heart diseases (NRVHD) are largely degenerative or bicuspid aortic valvular disease with rates of occurrence of clinically significant disease (moderate or greater severity) increasing with advancing age [2,34]. Nearly 40% of the world’s 30 million prevalent cases of NRVHD in 2017 resided in Western Europe and North America. The prevalence rates in these two regions were similar at approximately 1,500/100,000, far exceeding the 95/100,000 seen in SSA where most of the valvular heart diseases were due to RHD [2]. The low death rate from NRVHD in SSA of 0.5/100,000 compared to 6–10/100,000 in HIC is likely due to competing causes of death taking away inhabitants of SSA, before they are old enough to develop degenerative valvular heart disease. However, this could be due to some genetic difference in the occurrence of NRVHD, with less risk in blacks. Evidence to support this comes from the USA, where African Americans compared to Caucasians are at significantly lower risk of developing severe aortic stenosis due to degenerative calcific disease or congenitally bicuspid valve disease [82], as well as degenerative mitral valve disease presenting for surgery [83].

### Congenital Heart Disease

Congenital heart disease is the most common etiology of heart failure among children in SSA [9]. In 2017, approximately 12 million people were living with congenital heart disease (CHD) worldwide, with nearly 3 million of these in SSA [2]. The incidence (birth prevalence) of congenital heart defects in HIC of the western world is around 8 per 1000 live births [34,84], compared to only 1.9 per 1,000 live births in Africa which was lowest of all regions in one systematic review [84]. The possibility that this low birth prevalence in Africa might be a reflection of poor ascertainment given lack of structured perinatal diagnostic capabilities is likely. The only two studies from SSA that were included in this systematic review were conducted without modern echocardiographic imaging.

Many CHD cases in SSA are diagnosed at an advanced stage of their natural history when complications and contra-indications for corrective surgical interventions have already ensued [14,85]. A few hospital-based studies with imaging capabilities have been conducted in SSA [9,14,85,86,87]. A major study of 534 CHD patients at a cardiovascular referral center in Mozambique showed that complications were already present in 29% of cases at time of diagnosis. Among patients with an abnormality that needed surgical correction or palliation, only 41% of those could be operated with surgery contraindicated because of severe pulmonary hypertension, severe malnutrition, cardiomyopathy, neurological deficit, and HIV infection [14]. In the Cameroonian study, CHD patients requiring surgery were on the waiting list for more than three months before operation, and around 10% of patients died before their scheduled surgery, with surgical procedures completely paid for by not-for-profit organizations [85].

### Pericarditis

In SSA, the most common cause of pericarditis is tuberculosis accounting for about 65–91% of cases of pericardial disease across studies [88,89,90], compared to HIC of western world where about 80% or more of pericardial effusions are idiopathic with tuberculosis accounting for only <5% of cases [91,92]. In a recent systematic review that included 36 studies from SSA, tuberculosis was the most frequent cause of pericardial diseases in both HIV-uninfected and HIV-infected populations, followed by malignancies and rheumatological diseases [88].

Overall mortality rate from tuberculous pericarditis is high with one study reporting 26% during the six-month course of anti-tuberculosis treatment, and higher in HIV/AIDS patients than in those without (40% vs. 17%), in patients drawn from 15 referral hospitals in SSA [89]. Appropriate treatment to reduce mortality and morbidity are still lacking [93].

### Cardiovascular Disease in People Living with HIV Infection

About 37 million people globally are living with HIV. Nearly 26 million (70%) of these are residing in SSA, despite the region having only 15% of the world’s population [94]. HIV patients have higher rates of CVD than uninfected subjects, likely because of a combination of traditional risk factors, HIV-related inflammation, opportunistic infections, and highly active antiretroviral therapy (HAART) [95,96,97]. Contemporary data depicts that most common clinical cardiac complications associated with HIV in SSA are pericarditis, cardiomyopathy, and pulmonary hypertension, all leading to heart failure. Contrarily in the western world, IHD, lipodystrophy, metabolic syndrome, and prolonged QT leading to sudden cardiac death, are the most commonly seen cardiovascular manifestations of HIV/AIDS [95,96,98,99,100]. The prevalence of these cardiac complications of HIV in HIC pre-HAART era was up to 40%. Post-HAART era, HIV-associated pericarditis and myocarditis have almost disappeared from HIC, while IHD has emerged one of the main causes of death and disability of patients on HAART. In SSA, the pre-HAART era prevalence of these cardiac manifestations of HIV/AIDS was up to 60% [95]. Other HIV/AIDS-related complications include cerebrovascular disease and vasculopathy leading to peripheral vascular disease [98]. Although HAART does not seem to alter the long-term outcome in patients with established myocardial and pericardial disease, it seems to protect patients from developing these complications if introduced prior to their onset [95].

HIV-related cardiomyopathy is more common with increased immunosuppression (low CD4 count) and higher HIV viral load [99]. With increasing use of HAART and advances in HIV treatment, life expectancy has increased nowadays in people living with HIV, and HIV has slowly become a chronic disease. This increase in life expectancy portends the risk of CVDs from age and traditional risk factors as well as HIV and HIV treatment-related risk factors [101]. Thus, a one-stop integrated approach of proactively screening for CVDs and their risk factors during attendance at HIV clinics might prove to be highly beneficial and economical.

### Endomyocardial Fibrosis

Endomyocardial fibrosis (EMF) is an idiopathic heart disease seen mainly in the tropical and subtropical regions of the world, being the leading cause of restrictive cardiomyopathy in SSA, and probably worldwide [16,102]. EMF is exceedingly rare in western world except in immigrants from endemic areas [103]. In Africa, the disease has been reported in Uganda, Nigeria, Ivory Coast, Egypt, Ethiopia, Congo, Mozambique, Kenya, Sudan, Zimbabwe, South Africa, Ghana, Zambia, Malawi, Senegal, and Tanzania [16]. The highest prevalence rates of EMF are observed in SSA ranging from 1–20% with clustering in Uganda and Mozambique, while prevalence rates of up to 1.5% have been reported in India, 3% in China, and 2% in Brazil [104,105]. EMF trends have been hard to measure reliably [16], but marked reduction seems to be occurring in certain regions of the world like India [106], and even in some regions of SSA like Nigeria [107]. Untreated EMF carries very poor prognosis with the mean survival from onset of first symptoms being two years [102]. Despite various exciting hypotheses, no definite aetiology for EMF has been identified [16,102,104].

### Pulmonary Hypertension

Pulmonary hypertension (PH) in SSA is associated with very high mortality [108,109]. Community-based prevalence data for PH are lacking in SSA. In the Pan African Pulmonary Hypertension Cohort (PAPUCO), prospective multinational registry of newly diagnosed de novo consecutive patients with PH, the differential diagnoses of PH in adults were 16% pulmonary arterial hypertension (PAH), 69% PH due to left heart disease, 11% PH due to lung disease and/or hypoxia, 2% chronic thromboembolic pulmonary hypertension, and 2% PH with unclear multifactorial mechanism. At six-month follow-up, 21% of adults with follow-up data had died [108]. Similar findings were seen in the Cameroonian study that screened 2194 patients via echocardiograms, and observed a crude prevalence of PH of 15.6%, with mortality during six months of follow-up of 28% [109].

In South Africa, 20% of adult patients presenting with de novo right heart failure had WHO Group 1 (PAH) with nearly a two-fold increased risk in women compared to men. In women, the three most common forms of PAH were idiopathic PAH (34%), PAH related to concurrent HIV infection (33%) and PAH related to connective tissue disorders (27%), predominated by scleroderma. In men, the dominant form of PAH was idiopathic PAH (60% of cases) followed by HIV-related PAH (23%). In both sexes, congenital heart disease accounted negligibly to PAH. This is in comparison to registries in Europe and North America, where the most frequent aetiology of PAH was idiopathic accounting for 30–56% of all cases of PAH, connective disease 15–30%, and congenital heart disease 10–23% [110]. The finding of higher rates of PAH in women in SSA is consistent with observations in high-income countries [81], but may have distinct drivers.

### Peripheral Artery Disease

About 118 million people worldwide were living with peripheral artery disease (PAD) in 2017, and about 6.1 million of these lived in SSA. Since 1990, the prevalence, incidence, and mortality have increased worldwide, with the steepest increases seen in SSA [2]. An excellent systematic review in SSA revealed a high burden of morbidity from PAD, diagnosed by an ankle-brachial index (ABI) of <0.9, where prevalence ranged from 3.1% to 24% of adults aged 50 years and older and 39% to 52% of individuals with known risk factors like diabetes and smoking [111]. The findings of community prevalence rates in SSA are similar to high-income countries [112]. Low rates of secondary prevention strategies against PAD have been observed in SSA, with only 10% to 22% of patients with an ABI of less than 0.9 receiving antiplatelet therapy, only 2–12% of patients receiving statin therapy, and only very few case series reported vascular surgical treatment of PAD [111]. The main risk factors highlighted for the SSA region were similar to the western world and included age, diabetes, hypertension, tobacco use, and dyslipidaemia [111,112]. Is remains uncertain how many patients with acute limb ischemia in SSA have access to revascularization or amputation.

### Aortic Aneurysms

Aortic dissection or rupture which usually complicate an aortic aneurysm are surgical and medical emergencies that are beginning to be described in SSA, where the absence of appropriate imaging facilities and cardiothoracic & vascular surgery, makes most of them often fatal [113,114,115]. The GBD 2017 showed that mortality from all aortic aneurysms has trended down worldwide including SSA where it dropped to 0.75/100,000, but the total number of deaths in SSA from aortic aneurysms as well as the percentage of deaths in relation to total deaths have increased. The highest death rates are still in HIC where the range from 4.15 in North America to 7.14 per 100,000 in Western Europe [2]. The incidence and prevalence of AAA are increasing in SSA, while decreasing in WW [116]. Aortic aneurysms occur about 15 years earlier in blacks compared to whites in the Southern African region [117,118].

Hospital-based studies in Zimbabwe and South Africa have shown that hypertension and syphilis are the most frequent risk factors in black Africans, while hypertension and IHD are the most prevalent risk factors in Caucasians [117], and half of aneurysms in black Africans are of non-atherosclerotic origin (mainly due aorto-arteritis) [118]. In high-income countries, risk factors of TAA which is largely a degenerative disease include age, male gender, all atherosclerotic risk factors, aortitis, connective tissue disorders like Marfan, Loeys-Dietz, or Ehlers-Danlos syndromes, bicuspid aortic valve, and Turner syndrome. Similar risk factors are associated with AAA with smoking being the strongest modifiable risk factor, while diabetes is protective of AAA [119].

## Impediments to Effective CVDs Management and Recommendations

### Inadequate health budget allocation with disproportionate prioritization

Most member States of the African Region of the World Health Organization are still spending far less than the target of allocating at least 15% of annual expenditure to health under the Abuja Declaration [120]. Also, budget allocations towards management of CVDs and other NCDs from governments, non-governmental and international organizations appear to remain skewed and disproportionately low, compared to communicable diseases, with <5% of the bulk of global health funding being allocated to NCDs despite the growing burden of these diseases in SSA [22,120,121]. Understandably, balancing meager resource allocations with the pressing demands of endemic communicable, maternal, neonatal, and nutritional diseases can be very challenging. Evidence from sociology studies suggests very strong lateral and vertical intergenerational family networks and reciprocal wealth exchanges from richer to poorer in SSA [122]. Since current evidence suggests strong association of some NCDs with wealth and urbanization [11,23,25], a CVD death in a wealthy urban dweller will engender strong adverse socioeconomic consequences within the family and community of the victim given the close dependent network, a kind of negative cascading multiplier effect.

### Inadequate healthcare infrastructure

Most SSA countries have insufficient health care systems and infrastructure to manage CVDs with strong evidence of very limited number of hospitals equipped with the provision of specialist cardiac services in this region, including shortage of medications [18,19,20]. Tertiary centers for advanced cardiac imaging and invasive cardiac procedures including cardiac catherization, PCI, cardiothoracic and vascular surgical interventions are very sparsely available in most of SSA, apart from South Africa [18,61,123]. Electrophysiological study and catheter ablation centers are present only in very few countries in SSA and 26% of countries surveyed in this region do not have cardiac electronic implantable device implanting services [19]. Access to these facilities – where they exist – is still heavily limited by poor transport infrastructure, low availability or lack of reliable emergency medical services (EMS), with patients on average taking several hours to several days before arriving at these referral centers [18]. These delays often result in acute myocardial infarction patients, for example, arriving at health facilities outside the time window for thrombolysis and primary PCI. South Africa is the only exception in SSA where interventional cardiology and electrophysiological services and interventional rates have been successfully developed to international standards [61]. There have been some encouraging improvements in provision and availability of echocardiography in the SSA region [17,108,124]. It is not uncommon for wealthy individuals and high-grade politicians in SSA to be evacuated to the western world for treatment of CVDs, while the poor who cannot afford such care remain and perish or languish with the ensuing complications. Every country in SSA should strive to have at least one large tertiary referral academic center for treatment of CVDs and an automated ECG machine and capacity for transthoracic echocardiographic in district secondary-care facilities to capture initial CVD diagnoses.

### Shortage of cardiac professionals

Another important impediment holding back the tackling of CVDs in SSA is scarcity of cardiologists. There is a very low proportion of physicians to population, with a majority of SSA countries having <5 physicians per 10,000 people [125]. In a recent survey, 18% of the sub-Saharan African countries did not have a registered cardiologist [19,126]. The scarcity of African-born cardiologists signifies that SSA relies heavily on volunteering cardiologists from overseas, who might have the highest motivation, goodwill, and interest to care, but might not have a full insight into the cultural, linguistic, and sociopolitical dynamics of the various SSA communities to deliver effective cardiovascular care. International and regional training partnerships should therefore be fostered in innovative ways. For example, short-term six-month to one-year fellowship programs and training for specific procedures such as cardiac echocardiography or pacemaker implantation for non-specialists, nurses or technicians may be better than having no specialized care at all. Some nurse-led CVD management initiatives have proven successful in SSA, which falls in line with task-shifting advocated by the WHO as a means of increasing the health workforce [124].

### Sparsity of health insurance systems and high cost of CVD management

NCDs, especially CVDs are very burdensome cost-wise to manage [34]. Data show that in the majority of countries in SSA, direct out-of-pocket payments as a share of total health expenditure are still >40%, and far above WHO 20% threshold level of the total health expenditure below which financial risk protection can be ensured, and thus leading to impoverishment [121]. The sparsity of health insurance systems in most of Africa where only about 15% of the 55 countries have national comprehensive health insurance schemes [125], is a serious handicap. Development of insurance systems should be encouraged in public health and financial planning within countries. An inclusive universal healthcare system with national-level health insurance scheme is probably better as it will avoid the poorest stratum of the population from being left behind. This will increase health funds from prepaid sources which can then be subsequently pooled to allay the burden on individuals and their families. Some strategies like compulsory taxes or contributions from employees, deductions from sales taxes, heightened tobacco taxes, deductions from currency transactions, and so on, have been shown to be effective in some countries in SSA [120,121]. The 10 “Best Buys” strategies for reducing the burden of NCDs in SSA have been highlighted [127].

### Paucity of statistical epidemiological data

Inadequacies of complete epidemiological data collection on CVDs in SSA have been highlighted, with lack of proper disease surveillance programs noted in some countries [5,11,12]. Poor ascertainment and capture of the true burden and trends of CVDs and their risk factors might lead to inaccurate and underestimated statistical data. In such scenarios, public health policymakers and stakeholders will be ill informed about the true burden of CVDs, which could skew resource allocations away from these conditions to other health priority areas. Governments, international organizations, and academic institutions should develop structured surveillance programs and seek funding for research projects in this area in order to capture vital statistics and the true morbidity burden from CVDs.

## Conclusions

The burden of CVDs and their risk factors is increasing in SSA with available projections suggesting that in a few decades from now, CVDs and other NCDs will overtake communicable diseases as the most frequent cause of death in this region, particularly due to CAD and stroke. The region is unprepared for this growing burden, as there is evidence of insufficient health care infrastructure and resources as well as serious deficiencies in number of cardiac professionals to combat CVDs in this region. This is compounded by skewed and disproportional budget and resources allocations towards NCDs, where the priority is still rightly towards CMNNDs. Setting up healthcare systems for management of CVDs is expensive given costly CVD diagnostics and interventions, as well as need for life-long use of expensive CVD medications. As victims of CVDs in SSA are significantly younger compared to their western world counterparts, this poses an additional risk to regional socioeconomic development and health systems sustainability. Primary prevention should therefore be the key strategy to reduce morbidity and mortality from CVDs in SSA, and high-level strategic planning and partnerships are recommended involving governmental, non-governmental and international organizations, professional societies and associations, as well as local stakeholders.

## Abbreviations

CHD: congenital heart disease

CMNNDs: communicable, maternal, neonatal, and nutritional diseases

CVDs: cardiovascular diseases

HHD: hypertensive heart disease

HIC: high-Income Countries

IHD: ischemic heart disease

NCDs: non-communicable disease

NRVHD: non-rheumatic valvular heart disease

RHD: rheumatic heart disease

SSA: sub-Saharan Africa
